# A Study on the Effect of Different Cementitious Materials on the Mechanical Properties and Microscopic Characteristics of Alkali-Activated Green Ultra-High Performance Concrete (GUHPC)

**DOI:** 10.3390/ma18092163

**Published:** 2025-05-07

**Authors:** Zhiling Liao, Wanwen Xue, Lin Liao, Ruiqing Hao, Litao Shen, Dongxia Cui

**Affiliations:** 1 Department of Underground Engineering, College of Mining Engineering, Taiyuan University of Technology, 79 Yingze West Street, Taiyuan 030024, China; lzlfight66@163.com (Z.L.); xuewanwen0843@link.tyut.edu.cn (W.X.); 2 Shanxi Transportation Technology R&D Co., Ltd., Taiyuan 030032, China; shenlitao_008@163.com (L.S.); 18735368330@163.com (D.C.)

**Keywords:** ultra-high performance concrete, green low carbon, alkali-activated materials, mechanical properties, microstructure

## Abstract

This study investigates the influence of various cementitious materials on the performance of alkali-activated green ultra-high performance concrete (GUHPC). Alkali-activated GUHPC was prepared by substituting cement, quartz powder, and limestone powder with slag powder and fly ash. The mechanical properties, durability, hydration products, and microstructure were systematically analyzed. The results demonstrate that, with a cement dosage of 264 kg/m^3^, the alkali-activated GUHPC incorporating 40% slag powder and 28% fly ash as cement replacements exhibited superior mechanical performance, achieving compressive and tensile strengths of 165.3 MPa and 7.7 MPa, respectively, after curing. The GUHPC displayed a dense internal structure with an extremely low porosity of 6.76%, along with superior impermeability and frost resistance compared to conventional UHPC. Slag powder exhibited high pozzolanic reactivity under alkali activation, enabling effective cement replacement. These findings provide valuable insights for the formulation of alkali-activated GUHPC.

## 1. Introduction

Ultra-high performance concrete (UHPC) is an advanced cementitious composite material characterized by ultra-high strength (>120 MPa), high toughness, exceptional durability, and long-term stability, making it highly suitable for civil engineering applications [1,2,3,4]. However, the characteristics of UHPC due to the absence of coarse aggregate, low water-cement ratio, and fast hydration reaction speed, both excellent material properties at the same time, easy to lead to large heat of hydration, shrinkage and cracking, and other problems, which limits its large-scale promotion [5,6]. The amount of cementitious material in UHPC is as high as 1200 kg/m^3^, and the amount of cement is 900–1100 kg/m^3^, which is about three times that of ordinary concrete. However, the degree of cement hydration is only about 40%, and most of the cement is filled in UHPC as an expensive filler [7,8]. The data show that the production of one ton of Portland cement releases about 0.82 tons of CO_2_, and the amount of CO_2_ produced by the production of Portland cement each year accounts for 74–81% of global CO_2_ emissions, which is equivalent to about 3.24 billion tons of CO_2_ emitted into the atmosphere each year [9,10,11]. The high cement consumption in UHPC results in resource wastage and elevated carbon emissions, underscoring the importance of reducing cement content in UHPC formulations to promote sustainability. GUHPC is an eco-friendly binding material formed by alkali activation of aluminosilicate sources (e.g., fly ash, slag), offering high strength and corrosion resistance. It is widely used in construction (structural materials, rapid repair), transportation (road bases, railway sleepers), waste stabilization (hazardous waste encapsulation), refractory applications (fireproof coatings, kiln linings), marine engineering (chloride-resistant structures), 3D-printed construction, and military/emergency projects. Compared to conventional cement, it reduces CO_2_ emissions by 50–80% and enhances durability, though higher costs and evolving industry standards remain challenges.

Currently, the research on green ultra-high performance concrete (GUHPC) focuses on the preparation of ecological ultra-high performance concrete by replacing cement and other energy-intensive components with solid waste. For instance, Omar M. Abdulkareem et al. [12] used potassium hydroxide as an alkali activator and replaced 80% of cement with slag powder in UHPC, enhancing early compressive strength by 42% and 11% at 3 and 7 days, respectively. Shi et al. [13] replaced cement in UHPC with mineral powder silica fume, and the substitution amount reached 55%. The minimum content of cement was 370 kg/m^3^. Then sodium sulfate was used as the alkali activator to improve the activity of mineral powder silica fume and increase the pozzolanic reaction. The carbon emission and environmental impact index of GUHPC were about 40% and 35% lower than those of ordinary UHPC. Ding et al. [14] developed an optimized ultra-high performance concrete (UHPC) formulation based on a modified Andreasen & Andersen particle packing model, incorporating appropriate amounts of non-reactive filler as partial cement replacement. When cement clinker content was reduced to approximately 280 kg/m^3^, the environmental and mechanical properties of UHPC can be well balanced. Guo et al. [15] Successfully prepared green UHPC (GUHPC) using 30% limestone calcined clay cement (LC^3^) as Portland cement replacement combined with 30% recycled fine aggregates substituting natural quartz sand, achieving comparable mechanical performance to conventional UHPC while verifying the feasibility of LC^3^ and recycled aggregates in GUHPC production. Du et al. [16] utilized off-specification fly ash (OSFA) for GUHPC preparation, attaining optimal performance with 20% OSFA and 40% slag content. The optimal mixture achieved a 28-day compressive strength of 121.4 MPa, a flexural strength of 27.1 MPa, and a toughness of 7.3 kN·mm, along with significant reductions in lifecycle cost, carbon footprint, and embodied energy compared to conventional UHPC. Soliman et al. [17] developed an eco-friendly ultra-high performance glass concrete (UHPGC) using crushed glass powder as a partial replacement for cement and quartz powder, achieving exceptional workability, mechanical properties (220 MPa compressive strength), and microstructural characteristics. Huang et al. [18] demonstrated that 54% limestone substitution for cement enhanced workability while achieving 170 MPa compressive strength at 56 days.

This study proposes the development of alkali-activated GUHPC through the systematic replacement of cement with industrial by-products (slag powder and fly ash) at varying substitution levels. Comprehensive experimental investigations will be conducted, including mechanical property tests, durability assessments, hydration product analysis, and microstructural characterization. The strength development mechanism and binder reaction processes in GUHPC will be elucidated through phase composition analysis, pore structure evaluation, and internal morphology characterization. This research is different from the previous research in that it reduces environmental pollution and reduces the manufacturing cost of GUHPC while ensuring the excellent performance of GUHPC. The research aims to establish an optimized methodology for producing high-performance alkali-activated GUHPC, providing fundamental references for further development of advanced GUHPC systems through alkali activation technology.

## 2. Materials and Methods

### 2.1. Raw Materials

Cement for Jidong ordinary Portland cement (P·O 52.5) with a density of 3.1 g/cm^3^ (Hebei, China); Fly ash for Dingnuo material company’s I fly ash, density 2.3 g/cm^3^ (Henan, China); Silica fume for Platinum Run new materials of 98 silica fume, density 2.2 g/cm^3^ (Henan, China); Ground granulated blast furnace slag powder for Taigang S95 GGBS, density 2.5 g/cm^3^ (Shanxi, Chian); Limestone powder for Dehang’s heavy limestone powder, density of 2.5 g/cm^3^ (Hebei, China). A 2000 mesh quartz powder as filler to join. Use of three kinds of quartz sand, with aggregate sizes of 0.106–0.15 mm (S3), 0.15–0.212 mm (S2), 0.212–0.425 mm (S1). The steel fiber is a cylindrical fiber with a length of 13 mm and a diameter of 0.2 mm, with a tensile strength of 2300 MPa. Admixtures include polycarboxylate-based powder superplasticizer, defoaming agent, and expansive agent. The chemical activator is potassium hydroxide. The mixing water is ordinary tap water. The chemical composition of the binder materials is detailed in Table 1.

### 2.2. Mix Proportions

**Figure 1:**
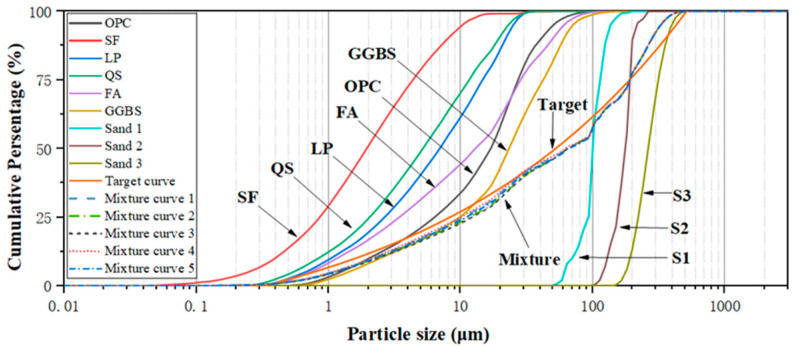
Particle size distribution of GUHPC raw material, target curve, and design curve.

The mix design of GUHPC follows the theory of the closest packing of particles and is based on the modified Andreasen & Andersen particle packing model [19,20]. Figure 1 presents the particle size distribution (PSD) curves of constituent materials in GUHPC, along with the target curve derived from the Andreasen model and the actual blended curves of the designed mixtures. The results demonstrate that the actual blended curves of the five GUHPC mix proportions show close alignment, with goodness-of-fit values to the target curve reaching 0.813, 0.806, 0.801, 0.819, and 0.811, respectively.

The mix design of green ultra-high performance concrete (GUHPC) based on the closest particle packing theory is presented in Table 2. To investigate the effects of varying binder material contents on the performance of alkali-activated GUHPC, 68% of the cement content in the conventional UHPC formulation was replaced with ground granulated blast furnace slag (GGBFS) and fly ash, while the proportions of other cementitious materials were systematically adjusted to develop GUHPC. To ensure optimal mechanical performance, potassium hydroxide (KOH) was incorporated as the chemical activator in the formulations. The mix proportions are summarized in Table 2.

### 2.3. Specimen Preparation and Curing

The manufacturing process followed these steps:

Dry mixing: Aggregates, binder materials, and admixtures were initially blended under low-speed mixing (300 rpm) for 90 s.

Alkaline solution preparation: The chemical activator (KOH) was dissolved in water, with 80% of the total mixing water introduced at this stage.

Primary mixing: The alkaline solution was added to the dry mixture under continued low-speed mixing (300 rpm) for 90 s, followed by a 30-s resting period to ensure homogeneous moisture distribution.

Secondary mixing: The remaining 20% of water was incorporated with low-speed mixing (300 rpm) for 180 s, then intensified to high-speed mixing (1200 rpm) for 120 s. Fiber incorporation: Steel fibers were gradually dispersed into the mixture during a final low-speed mixing phase (300 rpm) for 90 s.

**Figure 2:**
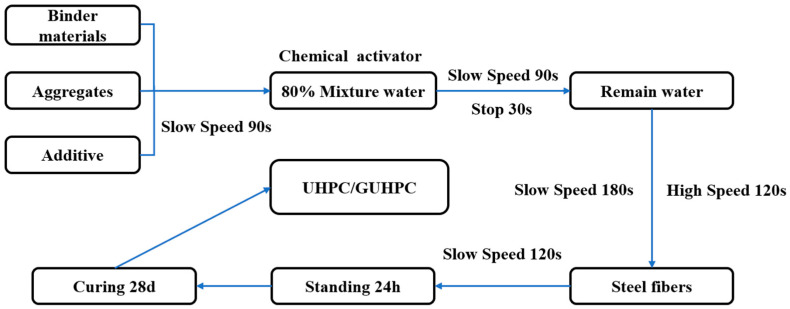
GUHPC preparation process flow chart.

The fresh mixture was then cast into pre-oiled molds, sealed with plastic film, and cured under ambient conditions (20 ± 2 °C) for 24 h before demolding. All specimens underwent standard moist curing in a controlled environment (temperature: 20 ± 2 °C, relative humidity > 95%) until 28-day testing. A detailed process flowchart is provided in Figure 2.

The density of the prepared sample is 2.54 g/cm^3^, and the density test method is gravimetric method.

### 2.4. Testing Methods

#### 2.4.1. Mechanical Property Test

The compressive strength tests were conducted following the Method of Testing Cement Mortar Strength (ISO) (GB/T 17671-1999) [21]. Cubic UHPC specimens (40 mm × 40 mm × 40 mm) were axially loaded on non-formed surfaces using a servo-controlled testing machine with a constant loading rate of 1.5 MPa/s. The experimental results were accurate to 0.1 MPa. The number of test samples is six.

**Figure 3:**
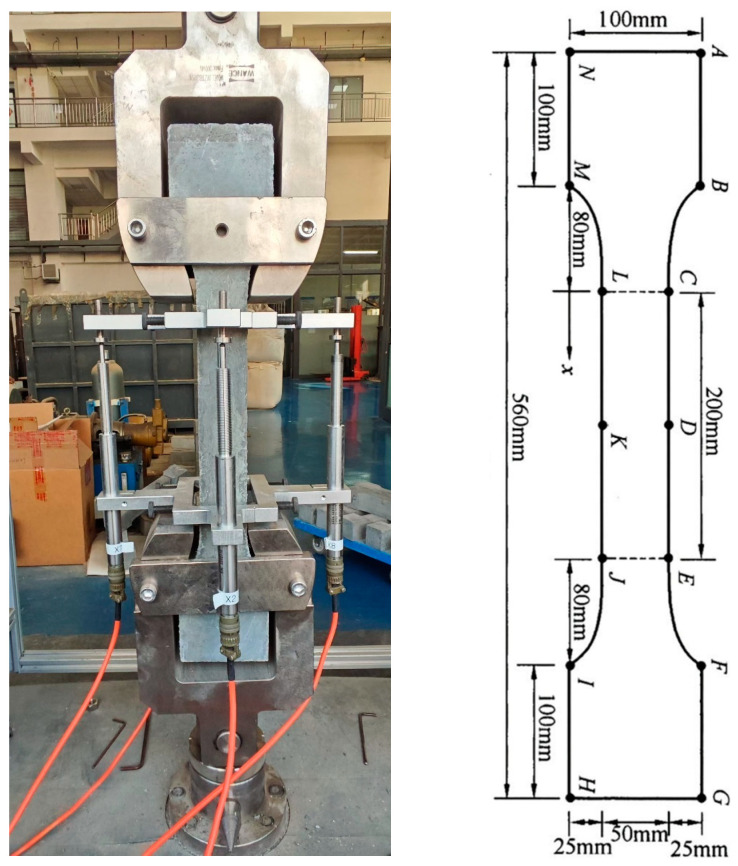
GUHPC tensile test setup.

Tensile strength evaluation followed Technical Requirements for Ultra-High Performance Concrete (T/CECS 10107-2020) [22]. Dog-bone specimens (100 mm × 100 mm × 560 mm) were tested under uniaxial tension at a controlled displacement rate of 0.2 mm/min. The experimental results were accurate to 0.1 MPa. The number of test samples is three. The experimental setup for tensile testing is illustrated in Figure 3.

#### 2.4.2. Chloride Permeability Test

The rapid chloride migration (RCM) method was employed to evaluate chloride ion penetration resistance, following these standardized procedures:

Specimen Preparation: Cylindrical samples (φ100 mm × 50 mm) were precision-machined from cured concrete.

Vacuum Saturation: Specimens underwent a 3-h vacuum saturation in a vacuum chamber, followed by a 20-h immersion in calcium hydroxide (Ca(OH)_2_) solution under saturated conditions.

Electrochemical Testing: Mounted specimens in rubber sleeves within test cells; Anodic chamber: 0.3 mol/L NaOH solution; Cathodic chamber: 10% NaCl solution (mass concentration); Applied DC voltage: 60 V (±0.1 V) for 96 h.

Post-Test Analysis: Split specimens axially using a compression testing machine; Sprayed fracture surfaces with 0.1 mol/L silver nitrate (AgNO_3_) solution; Measured chloride penetration depth via colorimetric analysis.

The chloride diffusion coefficient was calculated using Equation (1).

(1) $$D_{RCM}=\frac{0.0239\times \left(273+T\right)L}{\left(U-2\right)t}\left(X_{d}-0.0238\sqrt{\frac{\left(273+T\right)LX_{d}}{U-2}}\right)$$

#### 2.4.3. Freeze-Thaw Cycle Test

The frost resistance test adopts the freeze-thaw cycle test scheme designed in this paper. The basis for the temperature selection of the test scheme of the freeze-thaw cycle is designed according to the highest and lowest temperature in the region within one year. The time setting is based on the desire to make the freeze-thaw test more extreme and the conditions more harsh. The salt solution was prepared with 80% water and 20% NaCl. The sample test block and the control group C50 concrete test block were placed in a salt solution, frozen at −30 °C for 12 h, taken out and thawed for 3 h, placed at 60 °C for 6 h, taken out and cooled for 3 h, and then frozen again at −30 °C. Thus, for 24 h, a total of 30 cycles were done. Finally, the strength loss rate Δ*f_c_* and the mass loss rate Δ*W* are used as the indicators of the final freeze-thaw cycle resistance. ∆*f_c_* is calculated by Equation (2), and ∆*W* is calculated by Equation (3).

(2) $$\Delta f_{c}=\frac{f_{c0}-f_{cn}}{f_{c0}}$$

(3) $$\Delta W=\frac{W_{0}-W_{n}}{W_{0}}$$

∆*f_c_*: Compressive strength loss rate of concrete after N freeze-thaw cycles (%), accurate to 0.1%.
*f_c_*_0_: Compressive strength of reference concrete specimens (MPa), accurate to 0.1 MPa.
*F_cn_*: Compressive strength of concrete specimens after N freeze-thaw cycles, accurate to 0.1 MPa.
Δ*W*: Mass loss rate of concrete after N freeze-thaw cycles (%), accurate to 0.1%.
*W*_0_: Initial mass of concrete specimens before freeze-thaw cycling (g).
*W_n_*: Mass of concrete specimens after N freeze-thaw cycles (g).

#### 2.4.4. X-Ray Diffraction Analysis

X-ray diffraction analysis was performed using a Rigaku Miniflex 600 X-ray diffractometer (Japan). The instrument operated at 40 kV and 30 mA, with a scanning range of 5–90° (2θ) and a scanning rate of 2°/min. Prior to testing, samples were immersed in anhydrous ethanol for 24 h to terminate further cement hydration, followed by pulverization using an agate mortar grinder to produce fine powders with particle sizes < 45 μm.

#### 2.4.5. SEM Morphology Analysis

Microstructural characterization was conducted using a TESCAN MIRA LMS field emission environmental scanning electron microscope (Czech Republic). Before testing, the sample to be tested was first cracked into small pieces of about 1 cm in length and width and 1 cm in thickness, and soaked in anhydrous ethanol for 24 h to terminate the hydration of the cement, and then put into the drying oven to dry to constant weight. Gold spray treatment was carried out on the surface of the sample to ensure its conductivity. The test was carried out by using 10 kV accelerating voltage, placing the sample on the equipment, evacuating the vacuum, reaching the set vacuum level, increasing the voltage, raising the sample stage to a suitable height, looking for the sample position, selecting the shooting magnification, image scale, scanning the image, and saving the photo.

#### 2.4.6. Mercury Intrusion Porosimetry

Pore structure characterization was performed using a Micromeritics AutoPore V 9620 high-performance automated mercury porosimeter (USA). First, the sample to be tested was crushed, and a small piece of the middle position with a length of 1 cm and a thickness of about 1 cm was placed in anhydrous ethanol to terminate the hydration for 7 d, and then placed in a drying oven for 24 h. The sample was weighted, then transferred to the expansion meter. It was sealed and weighted, then placed into the low-pressure chamber. The low-pressure test was carried out, and the sample weighted again afterward. Next, the expansion meter, now injected with mercury, was placed into the high-pressure chamber. The high-pressure test was conducted, and the data analysis exported at the end. 

The statistical analysis methods used in this article include: descriptive statistical analysis, inferential statistical analysis, multivariate analysis and image processing, etc.

## 3. Results and Discussion

### 3.1. Mechanical Properties

#### 3.1.1. Compressive Strength

**Figure 4:**
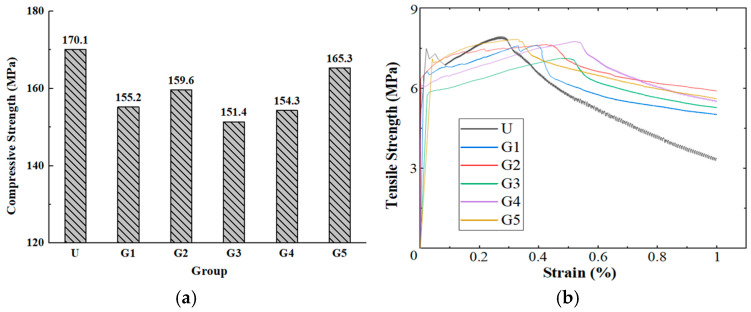
Mechanical properties test results: (**a**) GUHPC compressive strength test results; (**b**) GUHPC tensile stress-strain diagram.

Figure 4a presents the compressive strength test results of GUHPC. The data indicate that the alkali-activated GUHPC specimens exhibited lower compressive strengths compared to the control UHPC group. At the 28-day curing age, the compressive strengths of groups G1, G2, G3, G4, and G5 were 155.2 MPa, 159.6 MPa, 151.4 MPa, 154.3 MPa, and 165.3 MPa, respectively, corresponding to 91%, 94%, 89%, 91%, and 97% of the UHPC control group (U: 170.1 MPa). Among these, group G5 demonstrated the highest compressive strength, approaching that of conventional UHPC, while group G3 exhibited the lowest strength. This observation aligns with the earlier finding that group G3 had the poorest fit between its actual particle packing curve and the target curve in the mix design, confirming the significant influence of material packing efficiency on GUHPC strength development [23]. The reduced compressive strength of GUHPC compared to UHPC is primarily attributed to two factors. On the one hand, slower hydration kinetics, and the high replacement of cement with slag powder and fly ash in GUHPC decelerate the hydration process. At 28 days, the alkali-activated system produces less calcium silicate hydrate (C-S-H) gel compared to the conventional UHPC formulation. On the other hand, in interfacial microstructure, the UHPC matrix exhibits denser interfacial transition zones (ITZs) between aggregates/fibers and the cementitious matrix than GUHPC, owing to its optimized hydration products [24]. Group G2 showed strength improvement over G1 due to the replacement of limestone powder with slag and fly ash. While limestone powder primarily acts as an inert filler with limited reactivity and potential moisture absorption, slag and fly ash enhance C-S-H generation through pozzolanic reactions. Group G3’s substitution of quartz powder with slag and fly ash resulted in strength reduction. The fine quartz particles originally filled micropores as inert components, whereas the slower pozzolanic reactions of slag and fly ash failed to adequately fill the voids left by quartz removal. The superior performance of group G5 over G4 stems from the higher alkali activation potential of slag compared to fly ash. Slag demonstrates greater reactivity in alkaline environments, enabling more efficient C-S-H formation and thus prioritizing strength development in slag-rich formulations [25,26].

#### 3.1.2. Tensile Strength

Figure 4b illustrates the stress-strain curves of GUHPC under axial tensile loading. As shown, groups G1, G2, G3, G4, and G5 achieved ultimate tensile strengths of 7.6 MPa, 7.6 MPa, 7.1 MPa, 7.7 MPa, and 7.8 MPa at strains of 0.39%, 0.43%, 0.50%, 0.51%, and 0.32%, respectively. The control UHPC exhibited an ultimate tensile strength of 7.9 MPa at a strain of 0.28%, indicating comparable direct tensile performance between GUHPC and UHPC. The tensile strength hierarchy aligns with compressive strength trends: G3 (lowest) and G5 (highest). In GUHPC, the content of fly ash in the G1 group was 24%, the content of fly ash in the G5 group was 19%, and the content of fly ash in the G2, G3, and G4 groups was 29%. The data reveal that higher fly ash content extends the strain hardening phase of GUHPC. Specifically, formulations with elevated fly ash proportions (e.g., G2–G4) demonstrate prolonged strain-hardening compared to G1 and G5, suggesting fly ash enhances ductility by promoting microcrack bridging and delayed failure mechanisms. G5, with 29% slag powder and 19% fly ash replacing cement (remaining binder composition matching UHPC), exhibits a stress-strain curve nearly identical to UHPC. This indicates that alkali-activated slag powder achieves a high degree of hydration, effectively compensating for mechanical strength loss caused by ultra-low cement content. The slag’s superior reactivity under alkaline conditions drives robust C-S-H gel formation, enabling GUHPC to approach UHPC’s tensile performance despite minimal cement usage [25,26].

### 3.2. Chloride Permeation Analysis

To test the durability of GUHPC, the rapid chloride migration coefficient was used to evaluate its resistance to chloride erosion, and the results are shown in Table 3.

From the results in the table, the alkali-excited GUHPC and UHPC prepared with different cementitious material dosages have the same order of magnitude of impermeability, which indicates that GUHPC and UHPC have the same dense structure with small porosity. The chlorine ion diffusion coefficient of the five groups reveals that G5 is the smallest with only 0.078, and the rest are slightly larger than that of the U group, and the G3 group is twice as large as that of the U group.

The reason group G is larger than group U may be due to the filler dilution effect resulting in changes in the microstructure of GUHPC [27]. The addition of alternative cement fly ash and mineral powder introduces particles that are slightly larger than that of the cement particles, the same in the case of incomplete reaction, these larger particles are less effective than cement in filling the pore spaces within the concrete. The G5 group has the smallest diffusion coefficient, the reason is that it has smaller porosity and finer pore structure than UHPC, and the dosage of mineral powder in the G5 group is higher, and the higher alkalinity of the concrete promotes the geopolymerization reaction of the mineral powder under the addition of KOH [28], which reduces the internal porosity.

### 3.3. Frost Resistance Analysis

To test the frost resistance of GUHPC, this paper adopts the freeze-thaw cycling test program under extreme conditions, the results of which are shown in Table 4.

The experimental results demonstrate that GUHPC maintains exceptional frost resistance under extreme freeze-thaw cycling conditions. The strength loss rates of UHPC, GUHPC groups (G1–G5), and C50 control concrete are 1.6%, 3.6%, 3.3%, 11%, 2.5%, 0.85%, and 37.4%, respectively, while the mass loss rates are 0.2%, 0.3%, 0.16%, 0.48%, 0.16%, 0%, and 2%, respectively.

The significantly higher strength loss rate of group G3 (11%) is attributed to its elevated porosity and poor pore structure, allowing salt solution infiltration during freeze-thaw cycles. This triggers reactions with unreacted silica, generating expansive products that degrade internal integrity. Excluding G3, GUHPC exhibits comparable strength/mass loss to UHPC, confirming its superior salt-frost durability. This performance stems from the synergistic effects of slag and fly ash, which enhance pore refinement and sulfate resistance through pozzolanic and geopolymerization reactions [29,30]. Notably, group G5 outperforms UHPC due to its denser microstructure (lower porosity) and optimized pore distribution, achieved via high slag content (29%) under alkali-activated conditions.

### 3.4. XRD

**Figure 5:**
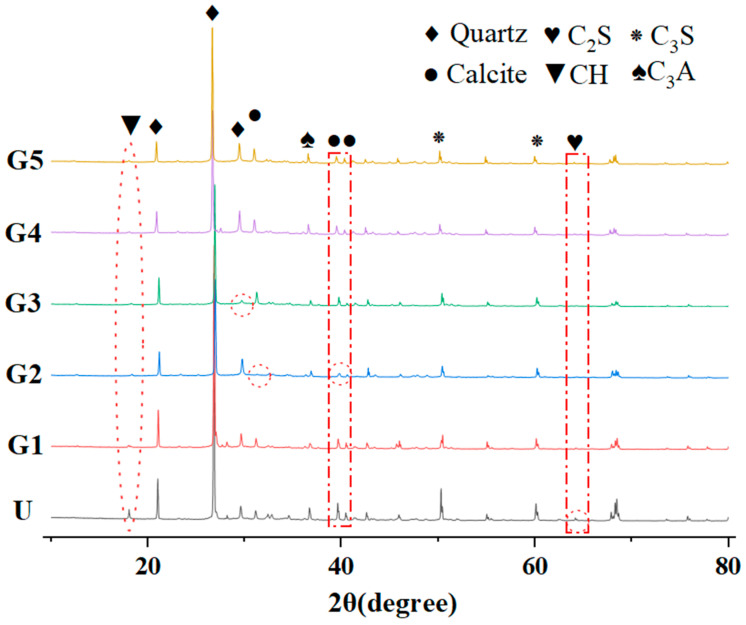
GUHPC 28 d X-ray diffraction pattern.

To study the connection and difference between GUHPC generation products, an X-ray diffraction analysis of GUHPC at 28 days was carried out. Figure 5 shows the X-ray diffractogram of GUHPC at 28 days.

From the figure, it can be observed that the main crystal phases in GUHPC are quartz, calcite, tricalcium silicate, dicalcium silicate, calcium hydroxide, and tricalcium aluminate, which is consistent with those in UHPC. The material phase detected in the diffraction peak at 18° was calcium hydroxide, with the peak of group U higher than that of group G and the lowest peaks in groups G5 and G3; the material phase at 21°, 27°, and 29.5° was quartz, with the lowest diffraction peak in group G3 at 29.5°; the material phase at 31.1°, 39.6°,and 40.5° was calcite, with the lowest peaks in group G2 at all the three angular positions; and the material phase in group G2 at 36.7°, 50.4° and 60.1°, 64.2°, tricalcium aluminates, tricalcium silicate, and dicalcium silicate were detected, respectively, with the highest diffraction peaks for the dicalcium silicate material phase in group U and almost none in group G. The diffraction of the dicalcium silicate material phase in group G was the lowest In all three angular positions.

The reason is as follows: the quartz phase detected at 29.5° may be from quartz powder composed of high-purity crystalline silica [31], while the quartz powder in group G3 is replaced by mineral powder and fly ash; group G2 has the lowest peak in calcite phase, and limestone powder does not participate in the reaction in the UHPC system [32], which makes us speculate that most of the calcite comes from limestone powder in the raw material, and this is also the same as group G2. The peak values of calcium hydroxide and dicalcium silicate were the highest and the lowest in groups U and G5, respectively, which indicated that most of the cement clinker in group U was not hydrated, but the peak value of calcium hydroxide was the smallest in the case of the higher dosage of mineral powders, which indicated that the addition of mineral powders facilitated the consumption of calcium hydroxide and the generation of the C-S-H volcanic ash reaction.

### 3.5. SEM

**Figure 6:**
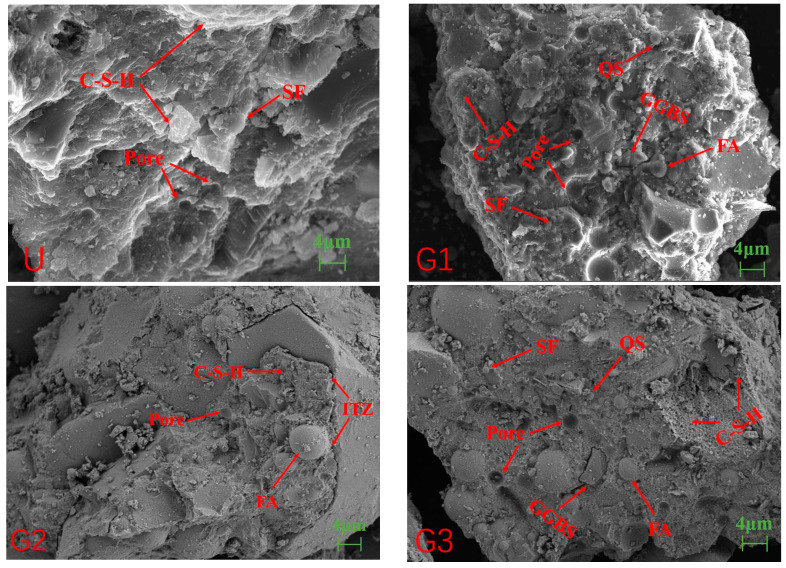

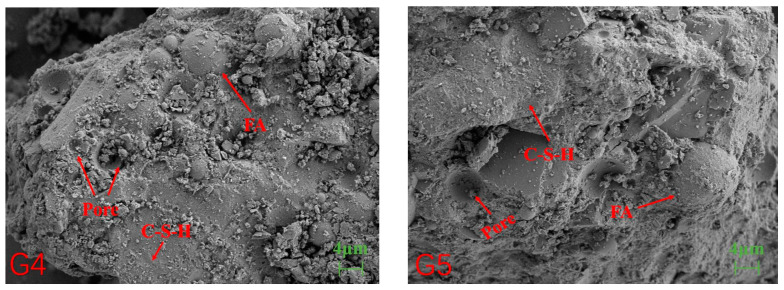
GUHPC micro-morphology diagram.

Figure 6 presents the micromorphology and microstructure of GUHPC observed via scanning electron microscopy (SEM). The UHPC exhibits a highly dense internal structure with minimal porosity, where calcium silicate hydrate (C-S-H) gel effectively encapsulates all components, including unreacted silica fume particles, forming a cohesive matrix. In G1, the microstructure remains well-structured with relatively low porosity, though an increased presence of spherical particles—corresponding to fly ash, quartz powder, silica fume, and slag powder—is evident. G2 displays complete encapsulation of constituents by C-S-H gel, with negligible porosity and a tightly bonded interfacial transition zone (ITZ) between hydration products and quartz sand; fly ash particles are visible, while slag powder is absent. G3 shares morphological similarities with G1 but notably contains Type I C-S-H, characterized by a less dense, reticulated structure compared to the typical Type II C-S-H observed in other groups. G4 resembles G2 in morphology but exhibits higher porosity, whereas G5 mirrors G1’s microstructure but contains unreacted fly ash particles embedded within the matrix.

Comparative analysis reveals that UHPC’s microstructure is slightly superior to GUHPC, with a higher density of hydration products explaining its enhanced mechanical strength. In GUHPC, the greater abundance of fly ash over slag is attributed to the higher reactivity of slag powder under alkali-activated conditions, leading to its preferential consumption in C-S-H formation. The presence of Type I C-S-H in G3 suggests delayed hydration kinetics in GUHPC systems, indicating potential for long-term strength development through continued pozzolanic reactions. The porous network of Type I C-S-H (common in alkali-activated systems) contributes to GUHPC’s marginally lower early-age strength compared to UHPC, while residual fly ash particles in G5 highlight incomplete activation, suggesting opportunities for optimizing alkaline dosage and curing conditions. This microstructural characterization aligns with prior mechanical and durability test results, providing critical insights into the composition-property relationships governing GUHPC performance.

### 3.6. Pore Structure

**Figure 7:**
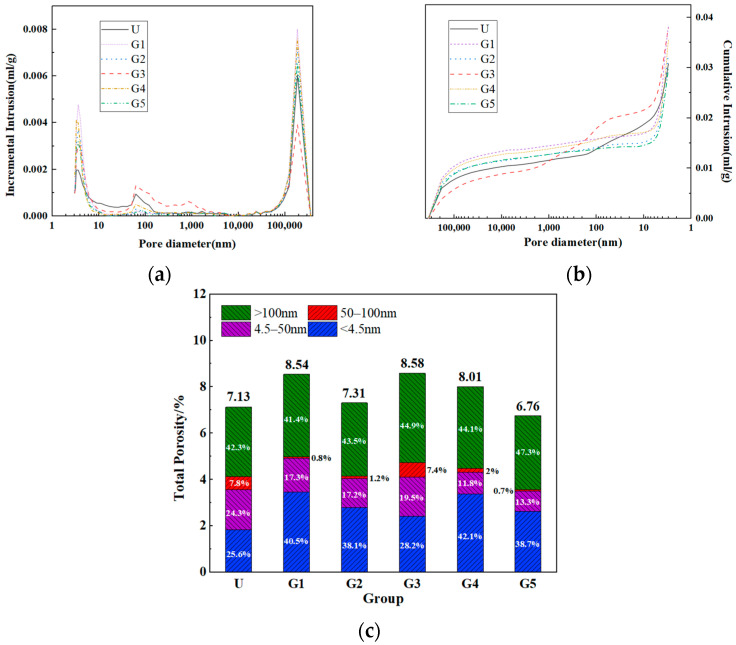
GUHPC Pore structure diagram: (**a**) Pore size development diagram; (**b**) Pore size distribution diagram; (**c**) Porosity diagram.

Figure 7 illustrates the pore size distribution and porosity characteristics of GUHPC, with pores categorized into four size ranges: Type I micropores (<4.5 nm), Type II mesopores (4.5–50 nm), Type III mesoscale pores (50–100 nm), and Type IV macropores (>100 nm). The pore structure analysis reveals the following:

As shown in Figure 7a, both UHPC and GUHPC exhibit a bimodal pore size distribution dominated by micropores and macropores, which account for 70–90% of the total porosity. Groups U and G3 display higher proportions of mesoscale pores compared to other groups. Figure 7b demonstrates similar pore development trends in UHPC and GUHPC, where groups U and G3 exhibit slower macropore development but faster growth of mesopores and larger pores beyond the mesoscale range. Figure 7c quantifies the total porosity as 7.13% (U), 8.54% (G1), 7.31% (G2), 8.58% (G3), 8.01% (G4), and 6.76% (G5), with G5 achieving the lowest porosity and G3 the highest. The mesopore proportions of groups U and G3 (7.8% and 7.4%, respectively) align with the pore size distribution patterns in Figure 7a.

The porosity results correlate directly with the mechanical strength, chloride ion penetration resistance, and frost resistance reported previously. Group G1 outperforms UHPC in impermeability and frost resistance due to its denser microstructure enabled by the multi-component cementitious system, where slag and fly ash physically fill pores caused by insufficient hydration products. The elevated mesopore content in group G3 stems from particle gradation deficiencies resulting from quartz powder replacement; finer inert quartz powder, which typically fills internal pores is absent, leaving voids under incomplete hydration. The higher porosity in group G2 compared to G5 arises from the non-reactive nature of limestone powder in GUHPC systems, which merely acts as a filler, whereas G5’s optimized formulation achieves superior pore refinement through synergistic material interactions. These findings confirm that pore structure optimization via tailored compositional design is critical for balancing GUHPC’s mechanical and durability performance.

## 4. Discussion

### 4.1. Optimization and Validation of Binder Replacement Strategy

Differences: Prior studies primarily focused on high-volume single industrial waste (e.g., 80% slag replacement [12] or 55% slag-silica fume composite replacement [13]), often overlooking the synergistic effects of multi-component binders.

Innovations: This study employs a combined replacement of 40% slag and 28% fly ash (total 68% cement substitution) with alkali activation (KOH) to optimize reactivity. At an ultra-low cement content (264 kg/m^3^), the compressive strength reaches 165.3 MPa, comparable to conventional UHPC (170.1 MPa), while achieving an exceptionally low porosity of 6.76%. Compared to literature [13] (370 kg/m^3^ cement) and [14] (280 kg/m^3^ cement), this further reduces cement usage without compromising performance.

### 4.2. New Insights into Microstructural Densification Mechanisms

Differences: Existing research emphasizes the pozzolanic effects of slag or fly ash [12,16] but lacks systematic analysis of inert fillers (quartz powder, limestone powder) in alkali-activated systems.

Innovations: Through XRD, SEM, and MIP analyses, this study reveals:

Quartz powder and limestone powder act primarily as physical fillers in alkali-activated GUHPC, non-reactive but critical for optimizing particle packing (e.g., G5 porosity: 6.76% vs. UHPC: 7.13%).

Slag exhibits higher alkali-activated reactivity than fly ash, forming denser C-S-H gels, explaining the superior strength of G5 (165.3 MPa). This complements findings in [23] on slag’s preferential reaction.

### 4.3. Enhanced Durability and Mechanistic Advancements

Differences: Most studies prioritize compressive strength [12,13,14], with limited focus on chloride resistance or freeze-thaw performance, especially under extreme conditions (e.g., salt freeze-thaw).

Innovations:

Chloride resistance: G5 shows a lower chloride diffusion coefficient (0.078 × 10^−12^ m^2^/s) than conventional UHPC (0.093 × 10^−12^ m^2^/s), demonstrating refined pore structure via multi-binder systems.

Freeze-thaw resistance: Under harsh salt freeze-thaw cycles (−30 °C to 60 °C), G5 exhibits minimal strength loss (0.85%), outperforming UHPC (1.6%). This validates the composite slag-fly ash system’s ability to mitigate salt crystallization damage, addressing gaps in [27,28]. 

### 4.4. Quantifiable Advancements in Low-Carbon Performance

Innovations: By combining 68% cement reduction with alkali activation, CO_2_ emissions are significantly reduced. Compared to [13] (40% lower carbon footprint), this study proves the feasibility of high performance at ultra-low cement content (264 kg/m^3^), providing new data for industrial-scale low-carbon applications.

### 4.5. Innovations

This study advances the field through multi-waste synergy + alkali-activated reactivity control, surpassing existing benchmarks in mechanical properties, microstructural density, durability, and sustainability. It specifically resolves the trade-off between strength and durability in ultra-low cement and UHPC, offering a superior solution for eco-friendly ultra-high-performance concrete engineering.

## 5. Conclusions

This study investigates the role of different cementitious materials in alkali-activated GUHPC through mechanical property testing, durability evaluation, and microstructural analysis of GUHPC prepared by replacing cement, limestone powder, and quartz powder in UHPC with slag powder and fly ash under chemical activation, yielding the following conclusions:

(1) GUHPC with ≤264 kg/m^3^ cement content can achieve excellent compressive and tensile strengths of 165.3 MPa and 7.8 MPa, respectively, using 40% slag powder and 28% fly ash as replacements;
(2) The multi-component cementitious system formed by slag and fly ash compensates for insufficient hydration products caused by cement reduction through physical filling, producing GUHPC with denser microstructure, lower porosity (6.76%), and enhanced durability;
(3) Under alkali activation, slag powder exhibits superior hydration reactivity compared to fly ash, generating greater quantities of hydration products (e.g., C-S-H gels) to improve mechanical strength, while increased fly ash content extends the strain hardening phase of GUHPC;
(4) Quartz and limestone powders function as inert fillers in the alkali-activated GUHPC system, physically occupying pore spaces without participating in hydration reactions.

## Figures and Tables

**Table 1:** The chemical composition of the binder materials.

Raw Materials	%Content by Weight								
	SiO_2_	Al_2_O_3_	Fe_2_O_3_	CaO	MgO	SO_3_	K_2_O	TiO_2_	LOI
Cement	19.41	5.97	4.32	61.12	2.83	3.27	0.86	0.17	1.42
FA	47.60	33.20	5.20	4.20	1.50	1.28	0.52	1.15	5.12
SF	98.1	----	0.03	0.11	0.14	0.02	0.04	----	1.48
GGBS	34.50	17.70	2.03	34.00	1.01	1.64	----	----	0.84
LP	0.05	----	0.13	55.41	0.10	0.15	----	----	42.54

FA represents fly ash, SF represents silica fume, GGBS represents Ground granulated blast furnace slag, LP represents Limestone powder, LOI represents Loss of ignition.

**Table 2:** The mix design of GUHPC.

	UHPC (kg/m^3^)	G1 (kg/m^3^)	G2 (kg/m^3^)	G3 (kg/m^3^)	G4 (kg/m^3^)	G5 (kg/m^3^)
Cement	820	264	264	264	264	264
SF	111	111	111	111	111	111
LP	111	111	----	111	111	111
QS	111	111	111	----	111	111
GGBS	----	275	330.5	330.5	219.5	330.5
FA	----	275	330.5	330.5	330.5	219.5
Sand1	550	550	550	550	550	550
Sand2	220	220	220	220	220	220
Sand3	330	330	330	330	330	330
Fiber	156	156	156	156	156	156
KOH	----	3.3	3.3	3.3	3.3	3.3
SP	5.8	5.6	5.6	5.6	5.6	5.6
EA	0.2	0.16	0.16	0.16	0.16	0.16
DF	1.7	1.5	1.5	1.5	1.5	1.5
Water	187	170	170	170	170	170

G1, G2, G3, G4, and G5 represents 1–5 groups of GUHPC, respectively.

**Table 3:** Chloride diffusion coefficient.

Mixture	U	G1	G2	G3	G4	G5
*D_RCM_* (10^−12^)	0.093	0.175	0.114	0.182	0.156	0.078

**Table 4:** Performance indexes before and after freeze-thaw cycles.

Mixture	U	G1	G2	G3	G4	G5	C50
$f_{c0}$ (MPa)	170.1	155.2	159.6	151.4	154.3	165.3	52.9
$f_{cn}$ (MPa)	167.3	149.6	154.4	134.7	150.5	163.9	33.2
$W_{0}$ (g)	647	639	638	625	639	641	586
$W_{n}$ (g)	646	637	637	622	638	641	574
$∆f_{c}$ (%)	1.6	3.6	3.3	11	2.5	0.85	37.2
∆W (%)	0.2	0.3	0.16	0.48	0.16	0	2

## Data Availability

The original contributions presented in this study are included in the article. Further inquiries can be directed to the corresponding authors.
